# Microenvironmental analysis of two alternating hosts and their impact on the ecological adaptation of the horned sumac gall aphid *Schlechtendalia chinensis* (Hemiptera, Pemphiginae)

**DOI:** 10.1038/s41598-019-57138-8

**Published:** 2020-01-16

**Authors:** Chao Wang, Ping Liu, Xiaoming Chen, Juan Liu, Qin Lu, Shuxia Shao, Zixiang Yang, Hang Chen, Kirst King-Jones

**Affiliations:** 1 Key Laboratory for Forest Resources Conservation and Utilization in the Southwest Mountains of China, Ministry of Education; Southwest Research Center for Landscape Architecture Engineering, National Forestry and Grassland Administration; Yunnan Province Engineering Research Center for Functional Flower Resources and Industralization, Southwest Forestry University, Bailongsi, Kunming City, Yunnan 650224 P.R. China; 2 Research Institute of Resource Insects, Chinese Academy of Forestry, Bailongsi, Kunming City, Yunnan 650224 P.R. China; 3 The Key Laboratory of Breeding and Utilization of Resource Insects of State Forestry Administration, Kunming City, Yunnan 650224 P.R. China; 4 Yunnan Forestry Technological College, Kunming City, Yunnan 650224 P.R. China; 5 Department of Biological Sciences, University of Alberta, G-504 Biological Sciences Bldg., Edmonton, Alberta, T6G 2E9, Canada

**Keywords:** Ecology, Plant sciences

## Abstract

The aphid *Schlechtendalia chinensis*(Bell) induces horned galls on their primary host *Rhus chinensis*(Mill). These galls serve as closed habitats to support thousands of aphids per gall. Ecological parameters inside a gall are unknown. In this study, we showed that the microclimate inside galls was reltively stable, with nearly 100% humidity and 30–50 lux light regardless of outside environmental conditions. Gall-residing aphids produce waste gas and honeydew. A gall contained 26 organic volatiles inside with acetic acid as the largest component. Honeydew is rich in sugars and may provide nutrients for microbial growth. However, no evidence for pathogenic microorganisms was found inside a gall. The acidic environment in a gall may curb microbial growth. On the secondary host, the moss *Plagiomnium maximoviczii* (Lindb.) T. J. Kop., the microclimate is unstable and humidity fluctuated at 45~100%, while light ranged from 150 to 500 lux on different environmental conditions. Aphid alternated in two different habitats, the gall generation increased from a single fundatrix to thousands of aphids, however, survival rate of the moss generation is less 3%. A comparison of the environmental traits between gall and moss revealed that a stable habitat with dark and moist is advantageous for aphid reproduction.

## Introduction

**Figure 1:**
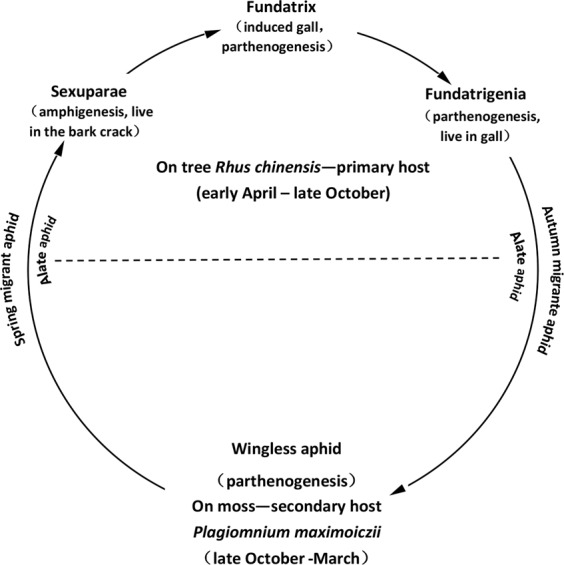
Life cycle of *Schlechtendalia chinensis*.

The horned sumac gall, found on the host plant *Rhus chinensis* (Mill), is induced by an aphid *Schlechtendalia chinensis* (Bell) and is the result of abnormal leaf wing rachis tissue growth. The aphid *S. chinensis* has a complex life cycle and exhibits cyclical parthenogenesis and gamogenesis by ovoviviparity on alternative hosts between tree and moss. During the life cycle of *S. chinensis*, aphids live alternately between its primary host tree, *R. chinensis* and secondary host moss, *Plagiomnium maximoviczii* (Lindb) T. J. Kop. Aphids migrate from galls to moss in October and remain there until March. From October to March, the aphids reside on the inner layer of soft moss where they reproduce one generation via parthenogenesis. The following spring in early April, the winged aphids generate when aphids turn into four instars, the winged aphid migrate from its winter host, the moss, to its primary host *R. chinensis*. Once aphids have reached the tree host, they deposit male and female sexuales in slits of branches. After mating, a female produces a single apterous fundatrix that feeds on the rachis wing and induces a closed gall, where fundatrix develop an adult aphid and reproduces three generations via parthenogenesis in it from May to October. At the end of October, the winged aphids migrate from broken galls to moss plant nearby under tree shade (Fig. 1)^1–3^. *Rhus* trees are distributed in most areas of China. However, the moss *P. maximoviczii* can only live in environments with high shade and moisture. Therefore, in nature, the horned sumac galling aphids are primarily distributed in regions around 25°~35°N latitude in China, where both *Rhus* trees and moss are present.

Although the aphid *S. chinensis* can live on both *Rhus* gall and moss hosts, the microenvironments of the two host species differ considerably, resulting in unique ecological and physiological challenges associated with each host for the aphid to adapt. While the environment on moss has been studied^4^, very little is known about the microenvironment inside the gall. Some researchers suggested that closed galls could provide a moderate effect on temperature buffering^5–7^. However, how to environmental change affect microclimate inside gall and inside layer of moss is still sealed. The gall is regarded as a structure that protects gall-inducing organisms from adverse environments, particularly desiccation^8–12^. However, dry environments in Australia do not result in more galls than in cooler, wetter environments^13^. Unlike some galling aphids induced gall that is for escaped from dry environment, horned gall by *S. chinensis* is only found environments with high moisture in China, specifically in regions with rainfall exceeding 1200 mm/year, relative humidity (RH) >80%. In spring, aphid *S.chinensis* migrate from its moss host to its tree host, which coincides with the start of the rainy season, gall generation occurred in rain season, whereas the fall migration from gall on *Rhus* trees to mosses also coincides with the beginning of dry season, moss generation lived in dry season. Galling aphids distributed in different regions could have their distinct migration behavior and ecological strategy to adapt adverse environment. A better understanding on the micro-ecological parameters in closed galls and the factors that drive aphid migration in autumn and spring will help to explain ecological adaptability of this aphid.

The gall of *S. chinensis* is a closed space in which thousands of individuals are housed during the later stages of aphid development^14^. Once the population density is high, the production of carbon dioxide and other waste gases levels will increase inside the gall, thus, raising the question as to how gas exchange with the exterior is regulated and how this affects the development of the aphid population. Moreover, gall-residing aphids produce substantial amounts of honeydew and at least one study shows that this is solved by absorption via the inner gall wall^15^. It is unclear, however, whether residual honeydew in the gall interior is still problematic, as it could provide a substrate for pathogenic microorganismal growth. Honeydew is a sugary liquid that may contaminate the colony both through physical exposure to a sticky substance as well as providing a breeding ground for pathogens^16–18^. However, despite high population densities within the gall we neither found evidence for microbial contamination on the inner gall surface nor transmission of pathogens among aphids. The apparent absence of disease and microbial growth suggested that defense mechanisms must have evolved to avoid or minimize disease vector transmission.

The objective of this study is to examine the dynamic microenvironmental changes that occur in aphid-produced galls and in living habitats on moss plants as well the consequences of these changes. Thus, we measured humidity, temperature, light intensity, concentration of volatiles in closed gall and acid concentration in the gall tissue. We also analysed other differences in environments associated with both host plants. This report discusses the parameters we observed between inside galls and in the layers of moss plants that have likely affected to aphid population growth. We also discuss the ecological reasons why aphids switch host plants.

## Results

### Structure of gall and volatile organic compounds inside gall

**Figure 2:**
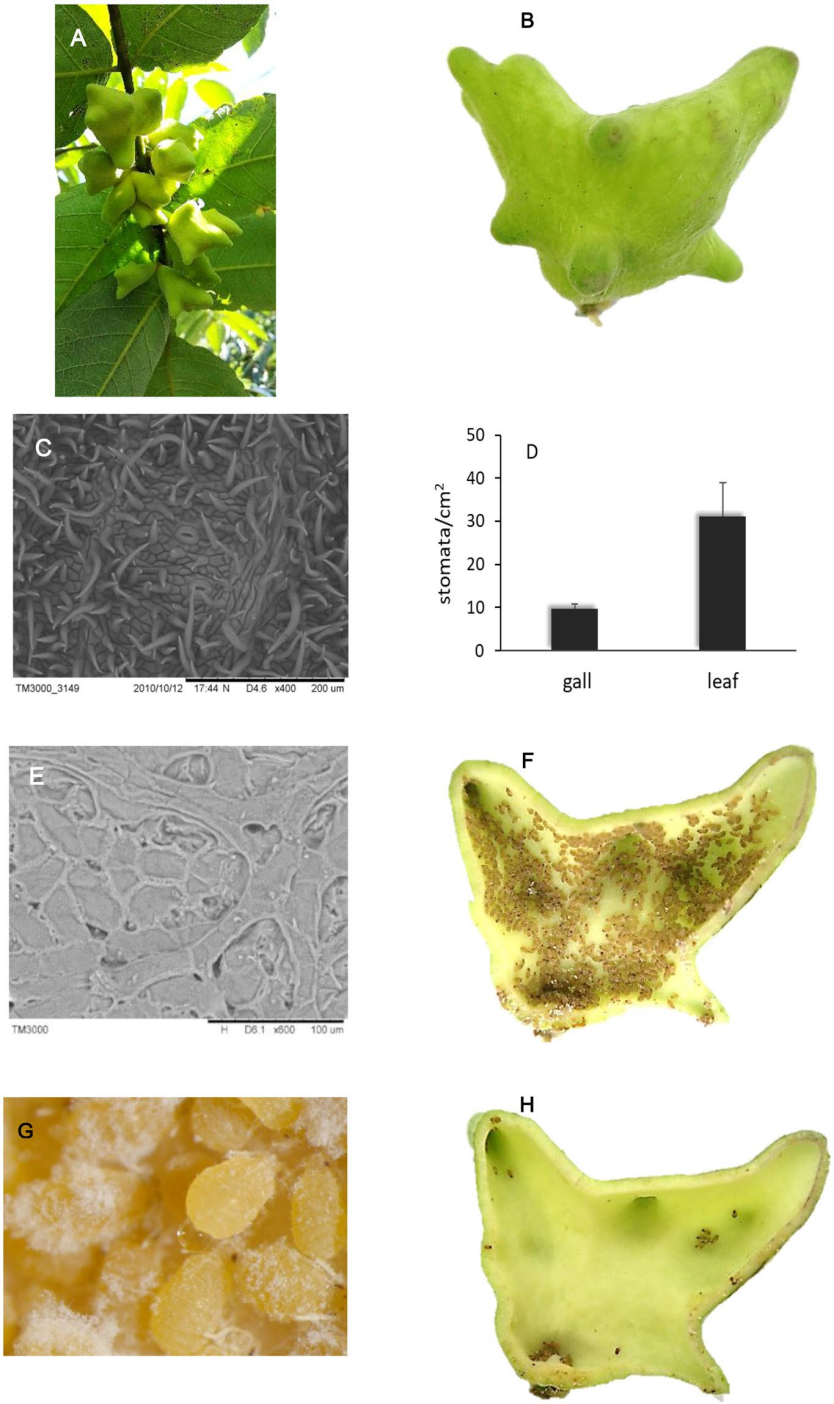
Physical parameter of horned gall and aphid excreted honeydew. (**A**) Galls grow on tree. (**B**) A single horned gall. (**C**) Stomata on outside surface of gall. (**D**) Stomata comparison between gall and leaf (error bars denote SD, n = 30). (**E**) Internal surface of gall. (**F**) Aphids living inside a gall. (**G**) An aphid is excreting honeydew in gall. (**H**) Clean interior wall surface of a gall.

Horned gall grew in clusters on branches of host tree and a horned gall is a closed space (Fig. 2A,B). On the gall wall, stomata are distributed in a dense array of spines on the external surface of galls (Fig. 2C). We found stoma density on the surface of gall was only about one-third of that found on leaves of the host tree (Fig. 2D), gas exchange inside and outside of gall could be impeded. Thus, the respiratory activity of large aphid populations could generate and accumulate substantial amount of gas metabolites. In this study, 26 volatile organic compounds were identified in addition to high CO_2_ levels, these volatiles include carboxylic acids, alkanes, olefins, alcohols, ethers, aldehydes, ketones and nitriles (Table 1). Among these 26 volatiles, acetic acid was the dominant compound, accounting for more than one third of all volatiles. Given the high level of acetic acid, the pH condition inside galls should be low. Indeed, pH of ground gall tissues was only 4.97 ± 0.02 (n = 10). There were also some crevices and pores on the interior gall surface (Fig. 2E), possibly important for the removal of honeydew. Although there were thousands of aphids excreting honeydew inside a gall, we dissected many galls and found that the interior of a gall was actually quite clean, with no sign of microbial contamination (Fig. 2F,H).

**Table 1:** Volatile organic compounds in gall (Mean ± SD, n = 3).

Volatile organic compounds	Relative amount(%)	Volatile organic compounds	Relative amount(%)
acetic acid	37.24 ± 2.19	hexanoic acid	2.57 ± 0.23
2-pentanone	1.26 ± 0.40	benzene, 1,3-dichloro	2.53 ± 2.06
toluene	4.76 ± 0.42	β-phellandrene	1.37 ± 1.91
2,4-dimethyl-1-heptene	0.30 ± 0.06	1-hexanol, 2-ethyl	1.31 ± 0.33
ethylbenzene	1.94 ± 0.11	heptane, 5-ethyl-2,2,3-trimethyl	0.43 ± 0.04
octane, 4-methyl	0.29 ± 0.01	undecane, 5,7-dimethyl	0.23 ± 1.36
*p*-xylene	3.74 ± 0.03	heptanoic acid	1.36 ± 0.85
*n*-butyl ether	0.24 ± 0.02	nonanal	0.83 ± 0.10
styrene	5.24 ± 3.37	benzyl nitrile	0.33 ± 0.02
heptanal	0.15 ± 0.01	octanoic acid	2.80 ± 1.50
*α*-pinene	5.40 ± 3.22	nonanoic acid	15.42 ± 7.08
benzaldehyde	0.70 ± 0.03	n-decanoic acid	1.25 ± 2.07
heptane, 2,4,6,6-pentamethyl	1.08 ± 0.76	Hexadecane	0.47 ± 0.122

### Temperature and humidity

**Figure 3:**
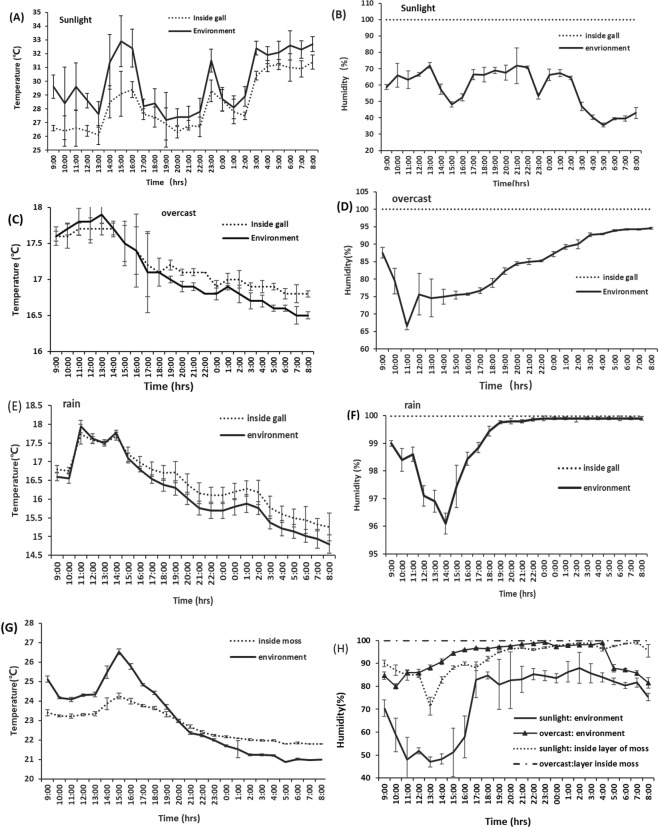
Temperature and relative humidity between environments, inside galls and in layers of moss plants(Error bars denote SD, n = 5). (**A**) Temperatures inside and outside galls on sunny day. (**B**) Relative humidity inside and outside galls in sunny day. (**C**) Temperatures inside and outside galls under overcast day. (**D**) Relative humidity between inside and outside galls under overcast day. (**E**) Temperatures inside and outside galls in rainy day. (**F**) Relative humidity inside and outside galls in rainy day. (**G**) Temperatures inside and above layers of moss plants. (**H**) Relative humidity inside and above layers of moss plants on sunlight and overcast days.

Galls are thought to provide physical barriers that protect galling insects from physical and biological hazards. A closed gall also produces a unique ecological environment for galling insects to live. Our data revealed that the temperature and humidity of the gall interior were regular difference from the conditions outside. When temperature was between 27~33 °C on sunny days, the interior temperature of the gall was 2~3 °C (1.58 ± 1.045, t = −7.73, *P* > 0.01) below the outside temperature (Fig. 3A). In contrast, when temperatures were below 17.5 °C on cloudy or rainy days, temperatures inside the galls were 0.1~0.5 °C (0.16 ± 0.11 on clouds day; t = 3.61, *P* > 0.01 and 0.33 ± 0.10 on rainy day, t = 7.05, *P* > 0.01) higher than the outside temperatures (Fig. 3C,E). The interior humidity of the gall remained almost constant at 100%, regardless of the environmental conditions (Fig. 3B,D,F). These data indicate that the gall structure serves as a buffer temperature fluctuation in both directions and suggests that the gall wall is an effective barrier to prevent evaporation. Similar to the gall interior, the temperatures inside the layer of moss also changed to a lesser degree compared with those above the moss layer. When temperature of the atmosphere was over 23 °C, the temperatures inside the moss layer registered 0.1~2.3 °C (1.22 ± 0.56, t = −6.0, *P* > 0.01) less, while at outside temperatures lower than 23 °C the temperatures inside moss layer were 0.1~1.0 °C (0.58 ± 0.30, t = 8.13, *P* > 0.01) higher, suggesting that interior layer of the moss plants can also serves as also slightly buffers temperatures (Fig. 3G), but less effective compared with galls. The humidity inside the layers of moss plants is more than 70% on different weather conditions, humidity inside layer of moss is 70~99% when environmental humidity ranged from 45% to 90% on sunny days, and humidity inside layer of moss is almost 99.9% when environmental humidity ranged from 80% to 99% on cloudy days (Fig. 3H). Indicated that moss has better moisture retention as gall.

### Light conditions inside galls and moss layers

The average light intensity outside the gall was 4932.5 ± 74.25 Lux on sunny days, but the average light intensity was 35.60 ± 7.78 Lux inside galls, the inside illuminance at less than 1% of this amount. On cloudy days, the average light intensity outside the gall was 572.47 ± 26.87 Lux. In comparison, the average light intensity was 40.8 ± 6.0 Lux inside the gall, representing 7% of that observed outside gall intensity. Despite much lower light intensity, light conditions remained relatively stable (35~40 Lux) inside galls under different weather conditions. On the other hand, the light intensity inside the layers of moss reached 150~500 Lux on cloudy and sunny days, which was much higher than those of inside galls (Table 2).

**Table 2:** Illuminance intensity (Lux) on two host (mean ± SD, n = 30).

	Outside gall	Inside gall	On moss	Inside moss
Sunshine	4932.50 ± 74.25	35.60 ± 7.78	4541.25 ± 2616.06	485.00 ± 187.50
Clouds	572.47 ± 26.87	40.80 ± 6.0	1765.00 ± 1011.68	148.33 ± 65.07

### Aphid population dynamics in galls and on moss plants

**Figure 4:**
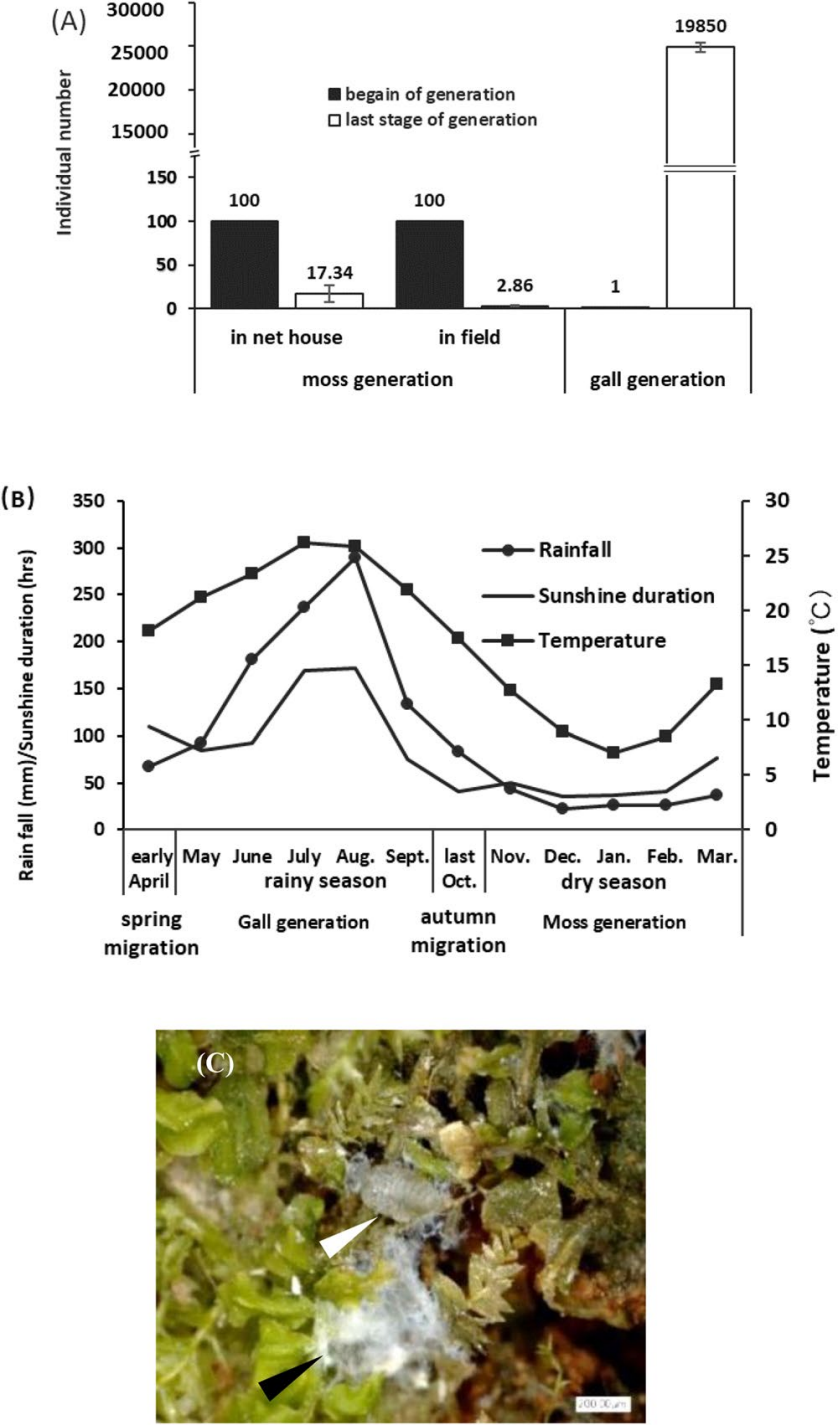
Population dynamic and environmental conditions between gall and moss. (**A**) Population dynamic inside gall and on moss (error bars denote SD, n = 30). (**B**) The weather conditions of gall generation and moss generation. (**C**) An aphid is secreting wax on moss (white arrows) and wax covered aphid on moss (black arrows).

There are significant differences in the fertility and population size of aphids that residing in galls and on moss plants. Inside a gall, a fundatrix generated a huge volume aphids via parthenogenesis in the subsequent three generations, which span seven months of life in the gall interior. On the secondary host moss, aphids resided for five months and produced one generation via parthenogenesis, however, the population size was dramatically reduced with survival rates being less than 3% (2.86 ± 1.10) in the open field and about 17% (17.34 ± 9.15) under the black shade cloth cage conditions (Fig. 4A). The ecological conditions under the black shade cloth cage and the open field site were somehow similar with the exception of higher humidity and lower light intensity, and overall more stable conditions under the cage, which are likely the reason for the higher aphid fecundity in the cage. Under natural conditions, aphids on moss plants live in the so-called dry season, whereas aphids in galls live in the rainy season (Fig. 4B).

## Discussion

The aphid *S. chinensis* has a complex life cycle that exposes the insect to different environments between the galls on sumac tree and its winter host moss. Our study revealed that aphid habitats on both *Rhus* trees and moss plants could provide a high humidity environment with low light conditions. Humidity and light intensity appeared to important environmental factors that influence aphid populations. The differences between these two hosts were higher humidity and lower light intensity in closed gall, and high humidity and low light intensity conditions in galls remained relatively stable under different weather conditions. On the other hand, humidity and light intensity fluctuated quite significantly on moss plants.

The microenvironment of high humidity and low light intensity inside a gall may provide benefits to the residing aphids. High humidity inside a gall could be primarily due to the respiration of large aphid populations in a closed system. Plant stomata are an important channel for exchange of gas and water vapor between gall interior and exterior environment. Stomate density on the gall surface is only one-third of that found on leaves of host trees, indicating that gall wall is less efficient for gas exchange and may have contributed to maintaining high moisture (100%) and gas accumulation inside the gall. In the a more open moss environment, humidity inside layer of moss fluctuated depending on the other environmental conditions but humidity is over 70%.

Overwhelming majority of aphids need light in their development and reproduction. For aphids living within a gall, most direct sunlight is blocked by the gall wall. Nevertheless, the gall interior has low but stable light conditions, with 35~40 Lux during day time under different weather conditions. On the other hand, the moss host grows in the shade, and illuminance in the moss varied from 150 to 500 lux during overcast or sunny days, thus exceeding the illuminance of the gall interior by at least 5~10-fold. Consistent with this, aphids secret a waxes substance while inhabiting on the moss host, presumably to cover their bodies with a wax layer that reduces exposure to light and water evaporation of aphid body (Fig. 4C). Galls play a role in temperature buffering: The gall wall can attenuate temperature changes inside the gall when environmental temperatures fluctuate. These observations confirm the idea that closed galls provide a moderate effect on temperature buffering^5–7^. A similar ability to buffer temperatures was also observed for the moss plants. However, temperature appears to not be of critical importance as moisture and light intensity for aphid development and reproduction, because the effect of temperature buffering is only limited in both galls and moss layers. In fact, the aphid *S. chinesis* is distributed between 25°~35°N latitude, and populations can survive in a wide range of temperatures.

There are crevices and pores throughout inner layer of a gall, which likely improve liquid wastes (such as water and honeydew) removal. Honeydew droplets are rich in sugars and may result in microbial growth if not removed. Kutsukake *et al*. (2012) indicated that honeydew in closed gall absorbed via the inner gall wall^15^. However, the residuum of honeydew is always on the interior surface of gall, which could breed microorganism. Despite high aphid population densities within the galls we neither found evidence for microbial contamination on the inner gall surface nor transmission of pathogens among aphids. An interesting possibility is that atmospheric conditions inside the gall contribute to maintaining a quasi-sterile environment to counter these hazards. Twenty-six volatile organic compounds were detected in galls, the main gas component among volatiles is acetic acid, which accounts for more than one third of all volatiles. Acetic acid and other volatile organic compounds such as β-phellandrene and octanoic acid may inhibit the growth of microorganisms^19–22^. In addition, high levels of tannic acid (>70%) were also found in gall tissue^23,24^, which may also help to curb microorganism growth^25,26^. Thus, it is possible that the combination of these microenvironmental parameters allow *S. chinensis* to thrive in a closed and crowded enclosure without any obvious microorganism contamination.

Many insects modify their environments directly, rather than merely choosing available sites that are already favorable, they constructed feeding or resting shelters such as galls^5^. The gall provides a shelter to aphids to avoid an adverse environment and protection against natural enemies. More importantly, this structure also provides a suitable microenvironment for growth and development of the aphid. In comparison, layers of moss plants are more labile to exposure to environmental extremes. The advantage of a gall can also be seen from aphid population expansion. Aphids reproduce three generations in a gall, resulting in thousands of offspring form from a single aphid fundatrix via parthenogenesis. In contrast, tens of thousands of aphids migrate from an individual gall to moss layers, where they reproduce for only one generation with less 3% of the initial population that can survive^2^ but bigger population sizes of aphids in moss layers under a in black cage. These results indicated that the low reproductive and survival rates of aphids in moss layers are likely due to high variation in microenvironments.

For most aphids, the evolution of complex life cycles may be driven by selection to segregate ecological resources such as food and refuge^27^. The complementary host growth hypothesis^27–33^ could explain why aphids migrate from tree to moss hosts because trees without leaves in Fall produce less energy and nutrients during winter, resulting in nutritional loss. Besides food resource, host alternation could escape from natural enemies and adverse environments, particularly in arid micro-habitats^5,9–12^. However, *S. chinensis* resides on the moss host during the dry season from October to March, while gall growth occurred in the rainy season from May to September, indicating that in this case the gall may not be a strategy to escape from desiccation since *S. chinensis* can undergo the dry season in a relatively unprotected habitat (on moss). Before the rainy season starts, aphids complete the spring migration from the moss to the tree host where they hide under tree bark until leaves sprout. The fact that aphids prefer living under tree barks to staying on moss plants indicates some reasons other than nutrients drive the spring migration. This suggests that one possible reason could be rainfall, as heavy rain will submerge the moss and reduce aphid survival. Additionally, there is period of time with higher light duration and intensity from May to September (Fig. 4B), which could be another reason for spring migration because the aphid prefers to a lucifuge condition to live. In the life cycle of *S. chinensis* on alternative two hosts, galls represent the principal habitat^31^, but the moss serves as a transitional host because *R.chinensis* is a deciduous tree, and as such loses leaves in winter, which would compromise nutrient availability for the gall and aphids. A comparison of the environmental traits between gall and moss suggested that aphids prefer a dark and moist habitat, while the gall provides additional safety from environmental hazards.

## Materials and Methods

### Gall collection

Field measurements were done on the fresh galls collected from the experimental site (N 28°06′E 104°22′, elevation 820 m) located in the natural distribution of gall, Yanjin county, Yunnan province, Southwest China. Climate condition: Annual average temperature is 16~18 °C; relative humidity is more than 80%; rainfall 1200~1300 mm. The 8-years old *Rhus* trees were used for gall production. The moss *P. maximoviczii* was planted on 3 cm fine grained soil under tree shade.

### Comparative examination on stomata between galls and leaf

To analyze and compare stoma condition between gall and leaf, we counted stoma numbers on the surface between gall walls and leaves by using a Scanning Electron Microscope (Hitachi TM3000, Hitachi Co, Japan). Thirty datasets were obtained randomly on stoma average numbers for both gall walls and leaves respectively.

### Measurements of volatile organic compounds

Measurements of organic volatiles were conducted at 10:00 a.m. on a sunny day in late August, During the measurement, a syringe with to a micro-pump was inserted into a gall grow on *Rhus* tree, volatiles from gall were pumped to the chamber by a micro-pump and collected by the sampling tube with adsorbents Tenax TA (0.2 g, 60–80 mesh, Perkin-Elmer) for 2 hours. Sampling tubes were sealed, stored 4 °C, and then analyzed by an ATD-GC-MS system (Automated thermal desorber-gas chromatography/mass spectrometry)^34^. All samples were analyzed in triplicates.

### pH measurement

The pH of fresh gall tissue was measured with a pH meter (HI2002-02, HANNA ITALY) after grinding. Ten fresh galls were used to pH measurements.

### Measurements of temperature and humidity in the gall and layers of moss

Gall experiments were performed in field in late August (when galls were fully developed). A minuscule hole (with a diameter 3~4 mm) was drilled through the gall and into a gall wall to allow the insertion of a miniature probe. The hole was sealed with wax after the probe was inserted into the gall, when parameters inside gall become stable, temperature and humidity were recorded by auto-thermohygrometers (Testo 635–2, Germany). Temperatures and humidity were recorded in parallel inside and outside a gall (5 cm away from the gall) during a range of conditions, including direct sunshine, overcast conditions and rain. Measurements were recorded every 10 minutes for 24 hours. Five samples were collected from five trees (one gall per tree). Measurements of microenvironmental parameters for the moss occurred in January. A miniature probe was inserted under a layer of the moss and another probe above the layer, and temperature and humidity were recorded every 10 minutes for 24 hours. Temperature and humidity of every hour were described at averaged value of six time recorded in one hour and plotted graph.

### Measurements of light penetrating into a gall

A hole was drilled into the gall wall as described in the previous section to allow the insertion of a miniature luminometer probe (Testo 150, Germany). The hole was then sealed with wax. Light inside gall was recorded 3 minutes after the light intensity became stable, The illuminance inside the gall during different weather conditions and determined light intensity outside the gall in parallel were recorded. Thirty galls were selected and measured randomly. A miniature luminometer probe was also inserted into a layer of moss to detect illuminance inside moss plants and another probe above the layer to determined light intensity above the moss in parallel. Thirty data were obtained randomly in 150 m^2^ moss.

### Recording dynamics of aphid population in the galls and on the moss plants

Prior to aphid migration to the moss plant, thirty gall samples were randomly collected, Aphids in individual galls were counted by opening the gall. To determine aphid population sizes on moss plants, we set up two experimental sites, one in black shade cloth cage (5 m × 4 m × 3 m) and one in the field. We planted moss *P. maximoviczii* on fine grains soil, in black shade cloth cage, illuminance allowed us to keep in the 100~200 lux range and humidity at 80%~90%. At the open field site, we measured illuminance at 100~500 lux and humidity at 50%~80% for host mosses that grew in the tree shade. At the beginning of autumn migration, 100 migrated aphids bred on an area of 50 cm^2^ moss. Prior to spring migration, we counted aphids again in the same area. Data from thirty samples were respectively obtained in both black tent and open field.

### Weather data

Weather data (temperature, rainfall and light) was obtained from the weather bureau of Yanjing county (horned sumac gall natural distribution area). Average temperatures, and rainfalls, and light intensities for the latest 30 years mean value was used for the analysis. The weather station is 15 kilometers away from experiment site.

### Statistical analysis

Statistical analysis was conducted using the IBM SPSS Statistics 19 software. T-test was conducted for temperature comparisons between inside gall, moss and environment.
